# Deficiency of Thrombospondin-4 in Mice Does Not Affect Skeletal Growth or Bone Mass Acquisition, but Causes a Transient Reduction of Articular Cartilage Thickness

**DOI:** 10.1371/journal.pone.0144272

**Published:** 2015-12-02

**Authors:** Anke Jeschke, Martin Bonitz, Maciej Simon, Stephanie Peters, Wolfgang Baum, Georg Schett, Wolfgang Ruether, Andreas Niemeier, Thorsten Schinke, Michael Amling

**Affiliations:** 1 Department of Osteology and Biomechanics, University Medical Center Hamburg Eppendorf, Hamburg 20246, Germany; 2 Department of Orthopedics, University Medical Center Hamburg Eppendorf, Hamburg 20246, Germany; 3 Department of Internal Medicine 3 and Institute of Clinical Immunology, University of Erlangen-Nuremberg, Erlangen 91054, Germany; University of Ulm, GERMANY

## Abstract

Although articular cartilage degeneration represents a major public health problem, the underlying molecular mechanisms are still poorly characterized. We have previously utilized genome-wide expression analysis to identify specific markers of porcine articular cartilage, one of them being Thrombospondin-4 (Thbs4). In the present study we analyzed *Thbs4* expression in mice, thereby confirming its predominant expression in articular cartilage, but also identifying expression in other tissues, including bone. To study the role of Thbs4 in skeletal development and integrity we took advantage of a *Thbs4*-deficient mouse model that was analyzed by undecalcified bone histology. We found that *Thbs4*-deficient mice do not display phenotypic differences towards wildtype littermates in terms of skeletal growth or bone mass acquisition. Since *Thbs4* has previously been found over-expressed in bones of Phex-deficient *Hyp* mice, we additionally generated *Thbs4*-deficient *Hyp* mice, but failed to detect phenotypic differences towards *Hyp* littermates. With respect to articular cartilage we found that *Thbs4*-deficient mice display transient thinning of articular cartilage, suggesting a protective role of Thbs4 for joint integrity. Gene expression analysis using porcine primary cells revealed that Thbs4 is not expressed by synovial fibroblasts and that it represents the only member of the Thbs gene family with specific expression in articular, but not in growth plate chondrocytes. In an attempt to identify specific molecular effects of Thbs4 we treated porcine articular chondrocytes with human THBS4 in the absence or presence of conditioned medium from porcine synovial fibroblasts. Here we did not observe a significant influence of THBS4 on proliferation, metabolic activity, apoptosis or gene expression, suggesting that it does not act as a signaling molecule. Taken together, our data demonstrate that Thbs4 is highly expressed in articular chondrocytes, where its presence in the extracellular matrix is required for articular cartilage integrity.

## Introduction

Osteoarthritis is a highly prevalent disorder characterized by loss of articular cartilage, a unique avascular and hypocellular tissue covering the joints [1,2]. The current treatment options are limited to surgical procedures, such as microfracture, osteochondral implantation, and, in the end-stage, total joint replacement [3,4]. In addition, autologous transplantation of articular chondrocytes after *ex vivo* expansion has emerged as a promising alternative therapeutic approach over the last two decades [5,6]. Since it is commonly accepted however, that any of these treatments only leads to formation of a fibrocartilagenous tissue with inferior quality compared to native articular cartilage, it is of utmost clinical importance to understand the molecular mechanisms controlling the function of articular chondrocytes. This may not only help to optimize ongoing tissue-engineering approaches, but also to identify potential molecular targets for pharmacological stimulation of matrix production by articular chondrocytes.

One potential explanation for the paucity of knowledge regarding specific regulators of articular chondrogenesis is the shortness of articular cartilage in mice, which hinders the identification of unexpected osteoarthritis phenotypes in genetically modified mouse models, unlike it was the case for other skeletal disorders [7]. For the same reason, it is extremely difficult to isolate primary articular chondrocytes from mice, which explains why many experiments related to arthritis have been performed with rib or epiphyseal growth plate chondrocytes. Regardless of these limitations several studies have been performed to identify specific markers of articular cartilage in mice, and there is an increasing number of mouse models displaying an arthritis phenotype [8–14]. Given the potential importance of identifying molecular differences between chondrocytes from articular and non-articular cartilage, we have previously performed genome-wide expression analysis with native tissues and cultured cells of porcine origin [15]. Here it was possible to separate articular and growth plate cartilage from the bone matrix and to culture a sufficient number of chondrocytes from both sources *ex vivo*. The major disadvantage of this approach was related to the use of porcine Gene Chips, which did not provide the same genetic coverage compared to the murine system.

Despite this limitation however, we made at least three important observations [15]. First, we identified common markers of cartilage, but also genes with specific expression in either growth plate or articular cartilage. Second, we found that the molecular differences between the two types of chondrocytes persisted after 10 and 20 days of *ex vivo* culture. Third, we defined 19 markers of articular chondrocytes, thereby raising the question, which of these are relevant regulators of articular cartilage integrity. Here we analyzed the physiological role of Thbs4, one of these previously identified markers, by studying the skeletal phenotype of a mouse deficiency model. We found that *Thbs4*-deficient mice do not display defects of skeletal growth or bone mass acquisition, but that articular cartilage thickness is transiently reduced.

## Materials and Methods

### 1. Mouse models

*Thbs4*-deficient mice on a C57Bl/6 genetic background were purchased from the Jackson Laboratories (#005845). Their genotyping was performed with the primers 5´-GGG TGG GAT TAG ATA AAT GCC TGC TCT-3´, 5´-GGA GAG AGA ATA GCA AGA TCA GCT C-3´, and 5´-AAC AAG CAA TGG AAG GCA GAC CCT G-3´, giving rise to a 412 bp and a 544 bp fragment for the wildtype and mutant allele, respectively. To evaluate the impact of Thbs4 in the context of X-linked hypophosphatemic rickets, *Thbs4*-deficient mice were crossed with *Hyp* mice (C57Bl/6 genetic background), which were also obtained from the Jackson Laboratories (#000528). To induce experimental arthritis, *Thbs4*-deficient mice were crossed with transgenic mice expressing human TNFα (Tg197 on a C57Bl/6 genetic background), which have been described elsewhere [16]. Joint swelling of the foot paws was assessed between 5 and 10 weeks of age using a clinical score graded from 0 (no swelling) to 3 (severe swelling of toes and ankle) as described previously [17]. Grip strength was analyzed on wire (diameter of 3 mm) using a score from 0 (normal grip strength) to -4 (no detectable grip strength) as described [17]. All mice were fed ad libitum and housed in a regular light/dark cycle under SPF-conditions. Animal experiments were approved by the animal facility of the University Medical Center Hamburg Eppendorf and by the “Amt für Gesundheit und Verbraucherschutz” (Org529).

### 2. Cell culture

Primary murine osteoclasts were generated by differentiating bone marrow cells for 10 days with 1,25-dihydroxyvitamin D3 (10 nM) added for the whole period, and M-Csf (20 ng/ml) and Rankl (40 ng/ml) added from day 3 until day 10 [18]. Primary murine osteoblasts were isolated by collagenase digestion of calvariae from newborn mice and differentiated for 20 days in the presence of ascorbic acid (10 mM) and ß-glycerophosphate (50 μg/ml) as described [18]. Porcine synovial tissue was obtained from the right and the left knee of 6 weeks old minipigs. Digestion of the prepared synovial membrane was performed with 1 mg/dl collagenase type 1a solution (Sigma-Aldrich, Germany) for 60–75 min at 37°C. Isolated synovial fibroblasts were seeded and cultured in Synoviocyte Basal Medium (Cell Applications, USA) supplemented with 10% heat-inactivated Synoviocyte Growth Supplement (Cell Application, USA) at normal cell culture conditions. Conditioned medium of these cells was collected for 24 hours in basal medium. To isolate chondrocytes bone-cartilage cylinders were harvested from the medial and lateral condyle of knee joints of 6 weeks old minipigs. The cylinders were separated in articular and growth plate cartilage under the dissecting microscope. Chondrocytes were released by collagenase type la solution (Sigma-Aldrich, Germany) as described [15]. The cells were cultured in DMEM/Hams’F12 (Biochrom, Germany) supplemented with 10% (v/v) FBS (Lonza, Germany) at 37°C under an atmosphere of 5% (v/v) O_2_ and 5% (v/v) CO_2_. These experiments were approved by the animal facility of the University Medical Center Hamburg Eppendorf and by the “Amt für Gesundheit und Verbraucherschutz” (Org356).

### 3. Expression analysis

RNA from murine tissues, cultured murine osteoclasts and osteoblasts, or porcine chondrocytes and synoviocytes was isolated using the RNeasyMini kit (Qiagen, Germany). DNase digestion was performed according to manufacturer’s instructions. Concentration and quality of RNA were measured using a NanoDrop ND-1000 system (NanoDrop Technology, USA). For RT-PCR expression analysis, 1μg of RNA was reversed transcribed using SuperScriptIII (Invitrogen, Germany) according to manufacturer’s instructions. Predesigned TaqMan gene expression assays (Applied Biosystems, Germany) were used to quantify expression of all murine genes, as well as for the porcine genes *ACAN*, *FN1*, *COL2A1*, *COL10A1*, *ASPN*, *FRZB*, and *SDC4*. For the other porcine gene the resulting cDNA was used for a PCR reaction with gene-specific primers (*PRG4*: 5´-CAT CTC TCT TTG ACG GTG AGG G-3´ and 5´-GCT CCA TAG TGC AGA CTT TCT TGA-3´; *THBS1*: 5´-CCA GCA GCC GTT TCT ATG TTG T -3’ and 5´-cct atg tga cga gga tca tgc-3´; *THBS2*: 5´-gac gag ttt ggg tct gtg ga-3´and 5´-cca gcg tag gtt tgg tca ta-3´; *THBS3*: 5´-cag gta cga ctg ctg tgg ac-3´and 5´-ggc act gtg tca ttg cat cg-3´; *THBS4*: 5’-ATC CAG GCG ATC GAA ATT CTG-3’ and 5’-AGG TGT CCT ATC GCT GGT TCC T-3’; *THBS5/COMP*: 5’-GGA TGC CTG TGA CAA CTG TC-3’ and 5’-AAG GCC CTG AAG TCG GTG AG-3’, *MATN3*: 5´-ACC CAC GCG CCC TAT TCT-3´and 5´-CGA GTG GGT CTG GAG ATG GA-3´; *GAPDH*: 5’-CTT CGT CAA GCT CAT TTC CTG G-3’ and 5’-AGT CAG GAG ATG CTC GGT GTG-3’) and SYBR Green Master Mix (Applied Biosystems, Germany). *GAPDH* expression was used as an internal control. Relative quantification was performed according to the ΔΔC_T_ method, and results were expressed in linear form using the formula 2^-ΔΔCT^ for both RT-PCR assays.

### 4. Skeletal analysis

After sacrifice the dissected skeletons were fixed in 3.7% PBS-buffered formaldehyde for 18 hours at 4°C, before they were stored in 80% ethanol. All skeletons were analyzed by contact radiography using a Faxitron Xray cabinet (Faxitron Xray Corp., USA) to measure the length of the lumbar spine and femora. For bone histology, the lumbar vertebral bodies L3 to L6 and one tibia of each mouse were dehydrated in ascending alcohol concentrations and then embedded in methylmetacrylate as described previously [18]. Sections of 5 μm thickness were cut in the sagittal plane on a Microtec rotation microtome (Techno-Med GmbH, Germany). For articular cartilage histology, knee joints were processed in the same way. All sections were stained by toluidine blue and von Kossa/van Gieson staining procedures as described [18]. Histomorphometry was performed according to the ASBMR guidelines [19] using the OsteoMeasure histomorphometry system (Osteometrics Inc., USA). Articular chondrocyte apoptosis was assessed on 5 μm thin decalcified paraffin sections by *in situ* TUNEL technology (Roche, #11684795910) according to the manufacturer´s instructions.

### 5. Cellular assays

To assess proliferation, metabolic activity and apoptosis, porcine articular chondrocytes were seeded into 96-well plates at a density of 1.000 cells per ml. On the next day cells were incubated for 24 hours with human THBS4 (R&D Systems, #2390-TH) at different concentrations in serum-free medium or in serum-free medium mixed with an equal amount of conditioned medium from porcine synovial fibroblasts. BrdU incorporation, MTT conversion and Caspase 3/7 activities were determined with commercially available systems (GE Healthcare Amersham, #RPM250, Sigma Aldrich, #M2158, and Promega, #G8090, respectively) according to the manufacturer´s instructions. To analyze cell adhesion 96-well-microtest plates were coated with 5 μg/ml THBS4 or 5 μg/ml THBS5/COMP (R&D Systems, #3134-CP) at 4°C overnight. Porcine articular chondrocytes were washed twice with HBBS/C (Hank´s Balanced Salt Solution + 1 mM calcium chloride), pre-treated with tosylphenylalanyl chloromethyl ketone-treated trypsin (0.1 mg/ml) in HBBS/C, before trypsinization was stopped after incubation for 5 min at 37°C. The cells were then washed three times with HBBS/C and resuspended in HBBS/C containing 1% heat inactivated BSA. Were indicated, a monoclonal antibody to integrin ß1 (BD Biosciences, #552828) was added to a final concentration of 16 μg/ml and incubated with the cells for 30 min at room temperature. Coated wells were washed four times with HBBS/C before addition of 3 x 10^4^ cells per well. After overnight incubation at 37°C under an atmosphere of 5% (v/v) O_2_ and 5% (v/v) CO_2_ the number of attached cells was quantified. To assess the effects of THBS4 and/or conditioned medium from porcine synovial fibroblasts on gene expression, cells were treated for 6 hours, before RNA was isolated for qRT-PCR expression analysis.

### 6. Serum analysis of individuals with osteoarthritis

Patients with osteoarthritis were recruited from the Department of Orthopaedics at the University Medical Center Hamburg-Eppendorf. Patient selection was based on a careful clinical examination and history according to the pattern of osteoarthritis (OA). Only individuals with primary OA were included. OA secondary to any known cause as well as any other arthropathy were exclusion criteria. All patients had severe, symptomatic primary OA of at least one large joint of the lower or upper extremity (knee, hip or shoulder; index joint) with radiographic joint space narrowing to less than a residual 1/3, and the request for total joint arthroplasty as the primary reason for consultation. Patients with knee or hip OA without signs and symptoms of OA in one or more additional joints were considered to have a relatively confined local disease and were classified as mono-osteoarthritis (mOA). Patients who, in addition to severe OA of the index joint, displayed clinically obvious hand OA of multiple interphalangeal joints and further joints of the lower and or upper extremities were considered to be more severely affected on a systemic level and were classified as poly-osteoarthritis (pOA). As an approach to distinguish between individuals with a presumably rather small total volume of articular cartilage affected locally by OA (mOA) versus individuals with a comparatively larger total volume of articular cartilage affected at multiple sites (pOA), we used these clinical differentiation criteria to build two distinct subpopulations of OA from our routine inpatient and outpatient clinic. Serum concentration of THBS4 was quantified using the antibody-based detection kit (Qayee-Bio, China). After the blood draw, serum samples were kept at room temperature for a maximum of two hours before storage at -70°C until analysis without additional freeze-thaw cycles. All participants provided written informed consent. This study and consent procedure was approved by the local ethics committee (Aerztekammer Hamburg, PV4037).

### 7. Statistical analysis

All data presented in the manuscript were obtained from the analysis of littermates (n ≥ 5) and are presented as means ± standard deviations. Statistical analysis was performed using unpaired, two-tailed Student’s t test, and p-values below 0.05 were considered statistically significant.

## Results

**Fig 1:**
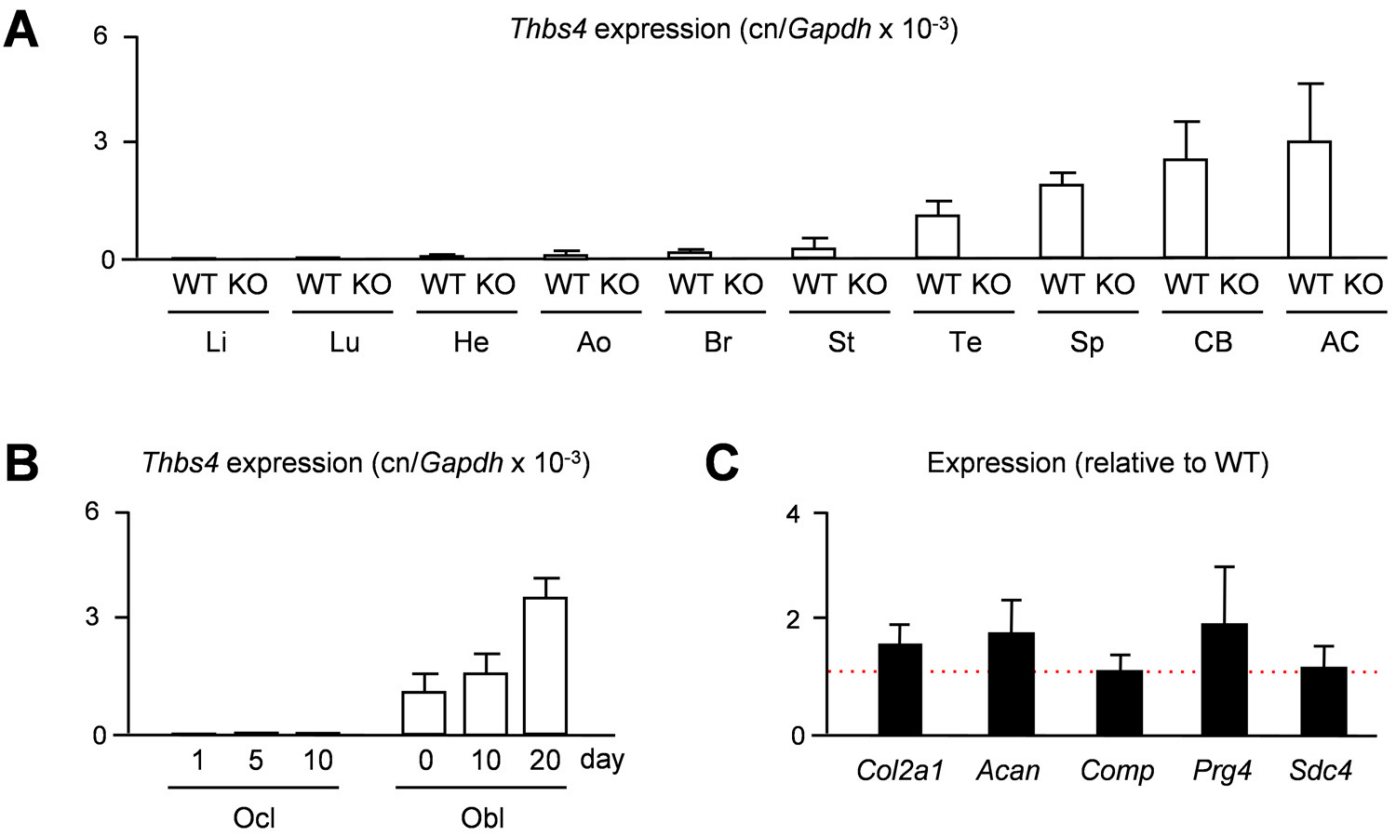
Murine *Thbs4* is highly expressed in skeletal tissues. (A) qRT-PCR monitioring *Thbs4* expression in various tissues of 15 weeks old widltype (WT) and *Thbs4*-deficient mice (KO). Li, liver; Lu, lung; He, heart; Ao, aorta; Br, brain; St, stomach; Te, tendon; Sp, spleen; CB, cortical bone; AC, articular cartilage. Bars represent mean ± SD (n = 4). *Thbs4* expression was undetectable in all KO tissues. (B) qRT-PCR monitoring *Thbs4* expression in primary osteoclasts (Ocl) and osteoblasts (Obl) from wildtype mice at different stages of differentiation. Bars represent mean ± SD (n = 3). (C) qRT-PCR monitioring expression of the indicated genes in articular cartilage of 15 weeks *Thbs4*-deficient mice. The dotted red line indicates the expression in wildtype littermates. Bars represent mean ± SD (n = 4).

Given the previously observed predominant expression of *THBS4* in porcine articular cartilage [15], we first addressed the question, whether the same is the case in mice. We therefore isolated RNA from different tissues of 15 weeks old wildtype and *Thbs4*-deficient mice to monitor *Thbs4* expression by qRT-PCR. Here we found, as expected only in wildtype mice, that *Thbs4* is highly expressed in articular cartilage, yet there was also strong expression in other tissues, such as tendon, spleen and cortical bone (Fig 1A). We additionally analyzed, which of the two bone remodeling cell types is the primary source of *Thbs4* expression and performed qRT-PCR with RNA from primary osteoclasts and osteoblasts at different stages of differentiation. Here we found that *Thbs4* was differentially expressed in osteoblast cultures, whereas *Thbs4* transcripts were undetectable in osteoclast cultures (Fig 1B). Since articular cartilage displayed the highest expression of *Thbs4* in wildtype mice, we additionally analyzed if *Thbs4*-deficiency would affect the expression of articular chondrocyte marker genes. Here we did not observe significant differences in the expression of *Col2a1*, *Acan*, *Comp*, *Prg4* and *Sdc4* between wildtype and *Thbs4*-deficient mice, suggesting that Thbs4 does not play a major role as a regulator of articular cartilage gene expression (Fig 1C). Nevertheless, given the high expression of *Thbs4* in skeletal tissues, we went on to study the skeletal phenotype of *Thbs4*-deficient mice.

**Fig 2:**
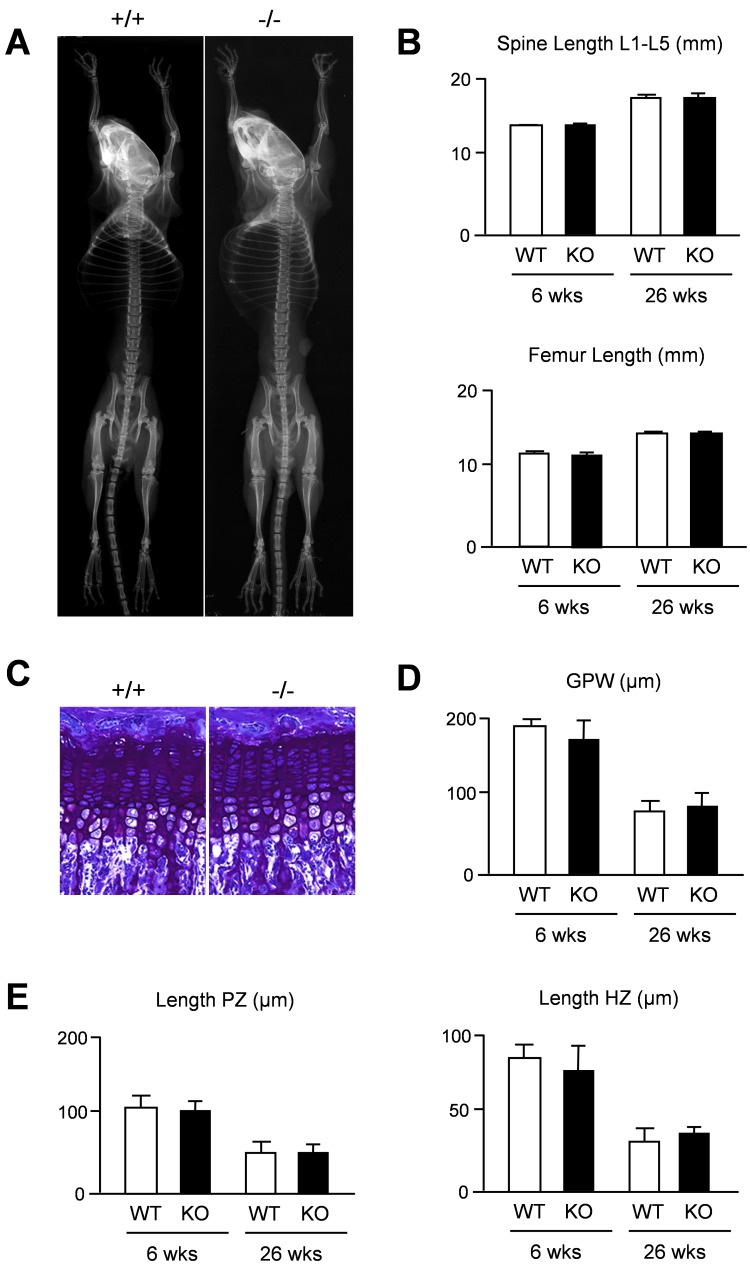
Intact skeletal growth in *Thbs4*-deficient mice. (A) Xray analysis demonstrates absence of gross skeletal abnormalities in 6 weeks old *Thbs4*-deficient mice. (B) Length of lumbar spine and femur in 6 and 26 weeks old wildtype (WT) and *Thbs4*-deficient (KO) mice. (C) Toluidine blue staining of the tibia growth plates from 6 weeks old wildtype (+/+) and *Thbs4*-deficient (-/-) mice. (D) Quantification of the tibial growth plate width (GPW) in wildtype (WT) and *Thbs4*-deficient (KO) mice at 6 and 26 weeks of age. (E) Quantification of the lengths of the proliferative zone (PZ) and hypertrophic zone (HZ) in the same sections. All bars represent mean ± SD (n = 6 per group).

**Fig 3:**
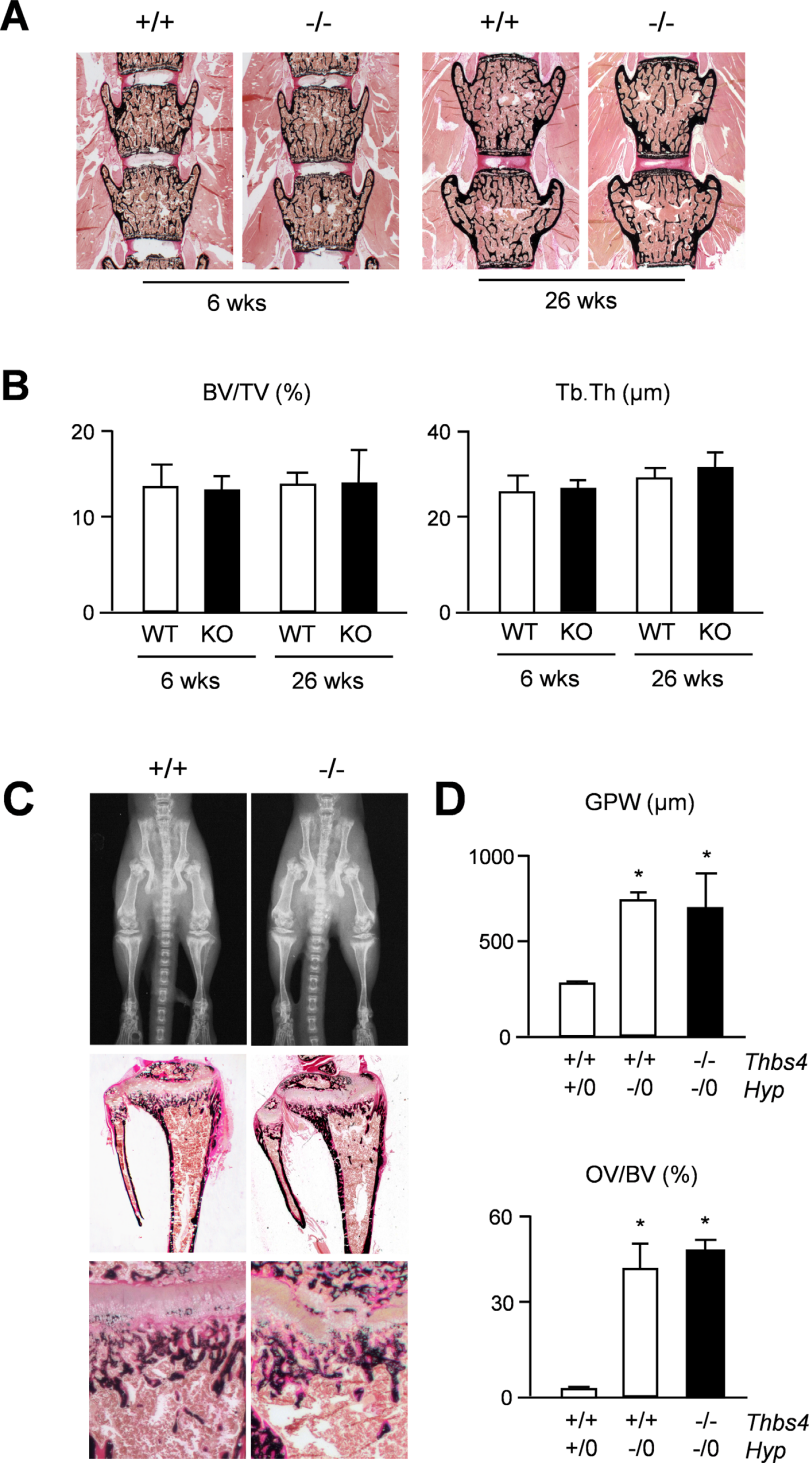
No impact of *Thbs4*-deficiency on bone mass or matrix mineralization on a wildtype or *Hyp* genetic background. (A) Von Kossa/van Gieson staining of spine sections from 6 and 26 weeks old wildtype (+/+) and *Thbs4*-deficient (-/-) mice. (B) Quantification of the trabecular bone volume per tissue volume (BV/TV) and trabecular thickness (Tb.Th.) in wildtype (WT) and *Thbs4*-deficient (KO) mice at both ages. Bars represent mean ± SD (n = 6 per group). (C) Xray analysis (top panels) and von Kossa/van Gieson staining of tibia (middle panels) or spine sections (bottom panels) from 6 weeks old Hyp mice with (+/+) or without (-/-) *Thbs4*. (D) Quantification of the growth plate width (GPW) in the tibia (top) or the osteoid volume per bone volume (OV/BV) from mice of the indicated genotypes. Bars represent mean ± SD (n = 5 per group). Asterisks indicate statistically significant differences towards WT controls (p<0.05).

We first applied Xray analysis, but failed to detect major differences towards wildtype littermates (Fig 2A). Likewise, quantification of spine and femur length did not reveal a significant difference between wildtype and *Thbs4*-deficient mice at 6 and 26 weeks of age (Fig 2B). We additionally applied non-decalcified histology of tibia sections to analyze for potential differences in the growth plate (Fig 2C). Histomorphometric quantification of the growth plate thickness did not reveal statistically significant differences between wildtype and *Thbs4*-deficient mice at 6 and 26 weeks of age (Fig 2D). Likewise, the lengths of the proliferative and hypertophic zones within the tibia growth plate were not affected by *Thbs4*-deficiency (Fig 2E). We additionally analyzed spine sections in the same groups of mice with respect to a potential bone phenotype (Fig 3A). By quantifying trabecular bone parameters we again found no difference between wildtype and *Thbs4*-deficient littermates (Fig 3B). Collectively, these data suggested that Thbs4 does not function as a physiologically relevant regulator of skeletal growth, bone formation or remodeling.

Another question to be addressed was based on previously reported findings in Phex-deficient *Hyp* mice, a model of X-linked hypohosphatemic rickets [20,21]. These mice display defects of skeletal growth and bone matrix mineralization, which are only partially explained by hypophosphatemia [22,23]. A genome-wide expression analysis revealed that *Thbs4* is markedly over-expressed in cortical bone of these mice, similar to *Fgf23*, whose increased expression in *Hyp* mice is known to cause their renal phosphate loss [24]. Since it was reasonable to speculate that a higher abundance of Thbs4 in the bone matrix could interfere with mineralization, we generated *Thbs4*-deficient *Hyp* mice and analyzed their skeletal phenotype. Here we found, as expected, that *Hyp* mice displayed a severe skeletal phenotype with pathological enrichment of non-mineralized osteoid, yet this pathology was not affected by *Thbs4*-deficiency (Fig 3C). Subsequent quantification of growth plate thickness and osteoid volume confirmed that the skeletal phenotype of *Hyp* mice was not significantly altered by additional *Thbs4*-deficiency (Fig 3D), thereby demonstrating that increased *Thbs4* expression by Phex-deficient osteoblasts is not involved in the pathogenesis of X-linked hypophosphatemic rickets.

**Fig 4:**
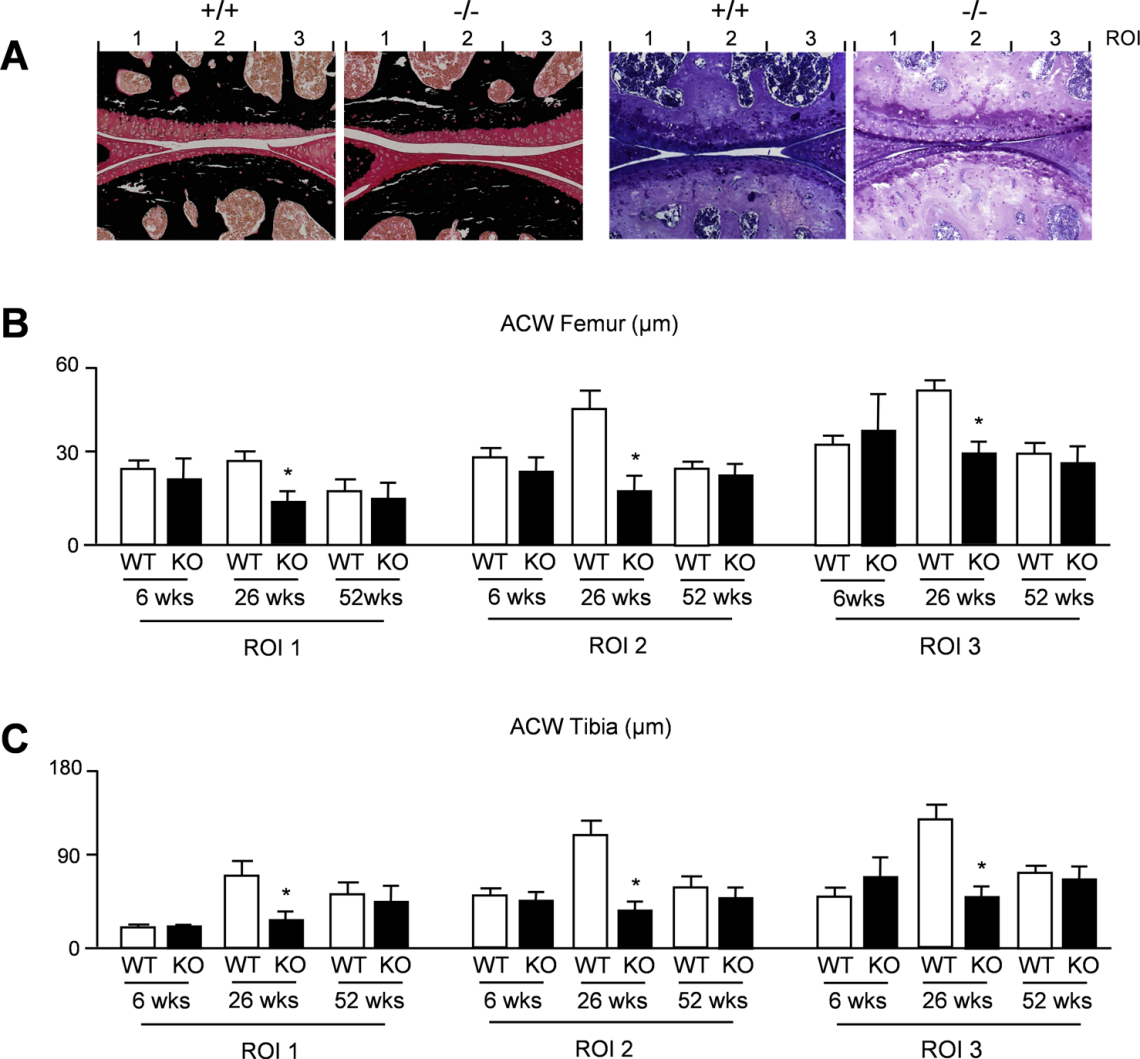
Transient thinning of articular cartilage in *Thbs4*-deficient mice. (A) Von Kossa/van Gieson (left panels) or toluidine blue staining (right panels) of articular cartilage from the knee joints of 26 weeks old wildtype (+/+) and *Thbs4*-deficient (-/-) mice. The three regions of interest for quantification of articular cartilage width are indicated. (B) Quantification of the articular cartilage width (ACW) in femora from wildtype (WT) and *Thbs4*-deficient (KO) mice at 6, 26 and 52 weeks of age. (C) Quantification of the articular cartilage width (ACW) in femur and tibia sections from the same mice. All bars represent mean ± SD (n = 6 per group). Asterisks indicate statistically significant differences between WT and KO (p<0.05).

**Fig 5:**
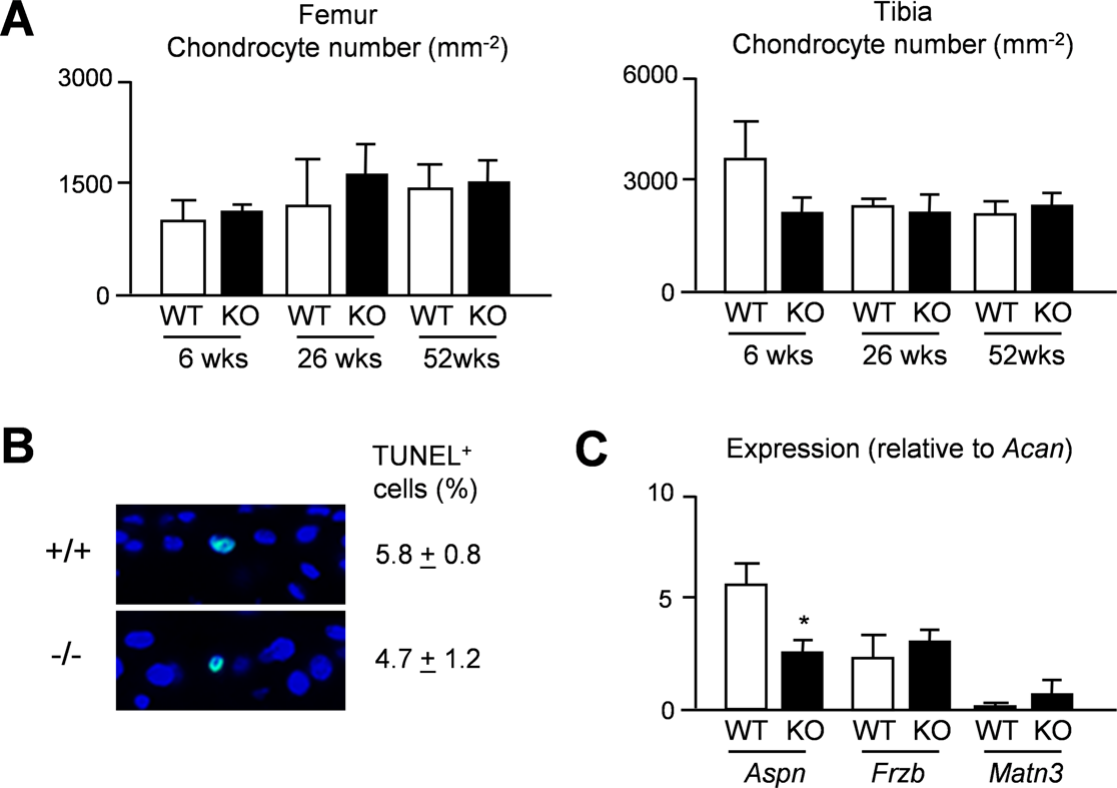
Chondrogenesis is unaffected in articular cartilage in *Thbs4*-deficient mice. (A) Quantification of the chondrocyte number per cartilage area in femur and tibia sections from 6, 26 and 52 weeks wildtype and *Thbs4*-deficient mice. Bars represent mean ± SD (n = 6 per group). (B) Representative images showing TUNEL-positive cells in articular cartilage from the femora of 26 weeks old wildtype and *Thbs4*-deficient mice. The percentage of TUNEL-positive cells is givenon the right. Values represent mean ± SD (n = 6 per group). (C) qRT-PCR monitioring expression of the indicated genes in articular cartilage of 26 weeks wildtype *Thbs4*-deficient mice. Bars represent mean ± SD (n = 4). Asterisks indicate statistically significant differences between WT and KO (p<0.05).

To assess the articular cartilage phenotype of *Thbs4*-deficient mice we analyzed sections from the knee joints of 6, 26 and 52 weeks old mice and determined the thickness of the articular cartilage layer in three different regions of interest (Fig 4A). Here we found that 26 weeks old *Thbs4*-deficient mice displayed significantly reduced articular cartilage thickness in all regions of interest, both in the femur (Fig 4B) and in the tibia (Fig 4C). This genotype-dependent difference was not observed at the age of 52 weeks, where the articular cartilage thickness was similar to 26 weeks old *Thbs4*-deficient mice in both genotypes. We also determined the number of chondrocytes per cartilage area in femur and tibia from the respective mice (Fig 5A). Here we did not observe statistically significant differences, and the same was the case for the percentage of apoptotic cells as assessed by TUNEL assay at the age of 26 weeks (Fig 5B). Finally, in an attempt to obtain a molecular explanation for the transient phenotype of *Thbs4*-deficient mice, we monitored expression of known osteoarthritis susceptibility (OAS) genes [25–28] in articular cartilage from 26 weeks old mice. Whereas *Gdf5* expression was not detectable by qRT-PCR in samples from either genotype, we found no significant changes between wildtype and *Thbs4*-deficient mice in terms of *Frzb* or *Matn3* expression (Fig 5C). Interestingly however, *Aspn*, encoding a small leucine-rich proteoglycan potentially inhibiting TGFß-dependent matrix synthesis [28,29], was expressed at lower levels in articular cartilage of 26 weeks old *Thbs4*-deficient mice.

To address the question, if *Thbs4*-deficiency would affect the severity of joint destruction in a mouse model of rheumatoid arthritis, we additionally crossed *Thbs4*-deficient mice with mice carrying a transgene causing over-expression of human TNFα [16,17]. Here we found that the presence of the transgene caused progressive joint swelling of the foot paws together with a decline in grip strength until the age of 12 weeks, yet *Thbs4*-deficiency did not significantly affect these two clinical scores (S1A Fig). When we histologically analyzed the knee joints at 12 weeks of age however, we found enhanced destruction of subchondral bone specifically in *Thbs4*-deficient TNFα-transgenic mice (S1B Fig). Taken together, these findings revealed that Thsb4 has a protective role in articular cartilage, although its deficiency does not affect the affect proliferation or apoptosis of articular chondrocytes.

**Fig 6:**
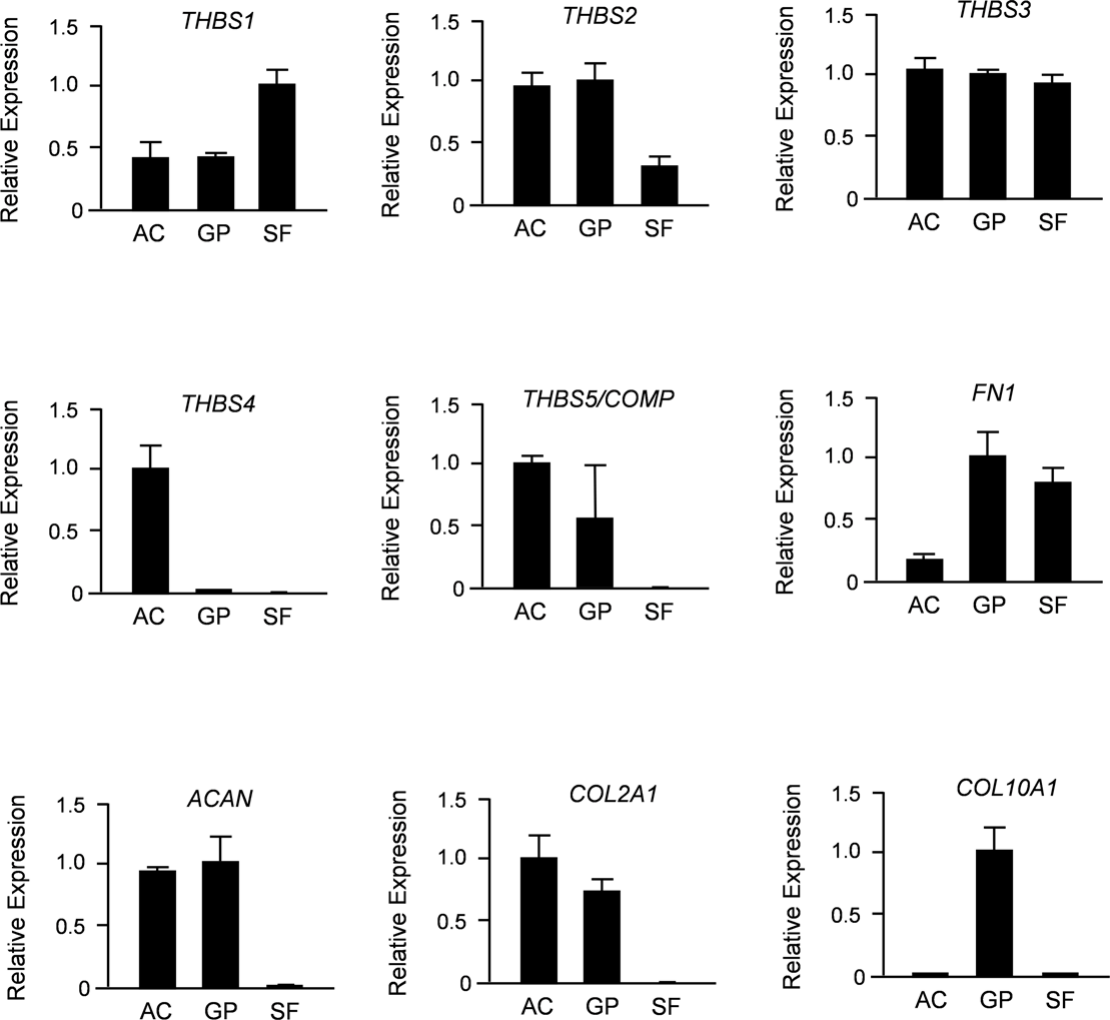
Porcine *THBS4* is specifically expressed in articular chondrocytes. Shown are the results of qRT-PCR expression analyses for all members of the *THBS* family, as well as markers for synovial fibroblasts (*FN1*), chondrocytes (*ACAN*, *COL2A1*) and hypertrophic chondrocytes (*COL10A1*). Primary cells (AC, articular chondrocytes; GP, growth plate chondrocytes; SF, synovial fibroblasts) were derived from 6 weeks old minipigs. Shown is the relative expression (after normalization to *GAPDH*) towards the cell type displaying the highest expression level. All bars represent mean ± SD (n = 3).

Since Thbs4 is only one member of a protein family, we next compared expression of all five thrombospondin-encoding genes in primary chondrocytes from articular and growth plate cartilage, as well as in synovial fibroblasts. To avoid any cross-contamination of these cell populations we again utilized minipigs, where the two types of cartilage, as well as synovial fibroblasts can be undoubtedly separated. Using qRT-PCR expression analysis we found that *THBS1*, *THBS2*, *THBS3* and *COMP/THBS5* were all expressed in both types of chondrocytes (Fig 6). With the exception of *COMP/THBS5*, we also detected their expression in synovial fibroblasts. In sharp contrast, *THBS4* expression was only detected in articular chondrocytes and not in any of the other cell types.

**Fig 7:**
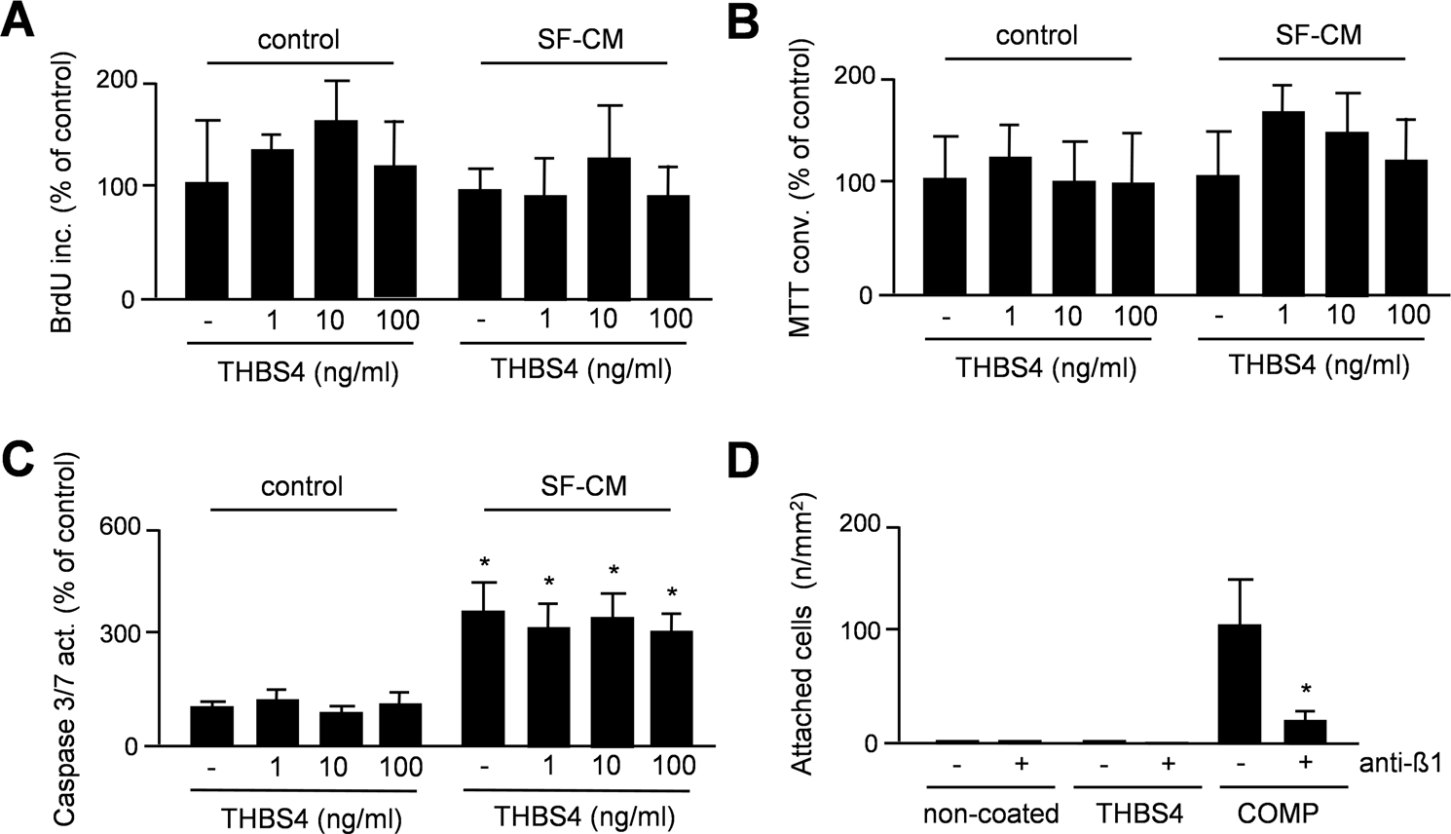
THBS4 does not affect the molecular behavior of porcine articular chondrocytes. (A) BrdU incorporation monitoring cellular proliferation of porcine articular chondrocytes in the presence of increasing concentrations of human THBS4 and/or conditioned medium from porcine synovial fibroblasts (SF-CM), as indicated. Bars represent mean ± SD (n = 5). (B) MTT conversion monitoring metabolic activity of porcine articular chondrocytes under the same conditions. Bars represent mean ± SD (n = 5). (C) Caspase-3/7 activity monitoring apoptosis of porcine articular chondrocytes under the same conditions. Bars represent mean ± SD (n = 5). (D) Cell attachment to non-coated plates or plates coated with THBS4 or THBS5/COMP in the absence or presence of a ß1-integrin antibody. Bars represent mean ± SD (n = 6). The asterisk indicates a statistically significant of the antibody (p<0.05).

To analyze a potential impact of THBS4 on the behavior of articular chondrocytes we also used porcine cells. More specifically, we assessed cellular proliferation (Fig 7A), metabolic activity (Fig 7B) and apoptosis (Fig 7C) over 24 hours in primary articular chondrocytes in the presence of increasing concentrations of human THBS4. As a control we performed the same assays with conditioned medium (50% final concentration) from cultured porcine synovial fibroblasts (SF-CM), and again co-administered increasing concentrations of human THBS4. Here we found that THBS4 did not cause a significant influence on any of the parameters. Interestingly however, while SF-CM did not affect proliferation or metabolic activity of the articular chondrocytes, it significantly increased the Caspase-3/7 activity, suggesting a pro-apoptotic influence, which was however unaffected by THBS4. Finally, since COMP/THBS5 has been shown to mediate chondrocyte attachment in an integrin-dependent manner [30], we analyzed if THBS4 would serve a similar function. To address this possibility we coated non-tissue culture plates with THBS4 or COMP, before adding porcine articular chondrocytes in the presence or absence of an antibody against ß1-integrin. After 24 hours we counted the adherent cells and found that they attached to COMP-coated plates in a ß1-integrin-dependent manner (Fig 7D). In contrast, we failed to detect adherent articular chondrocytes on THBS4-coated plates.

**Fig 8:**
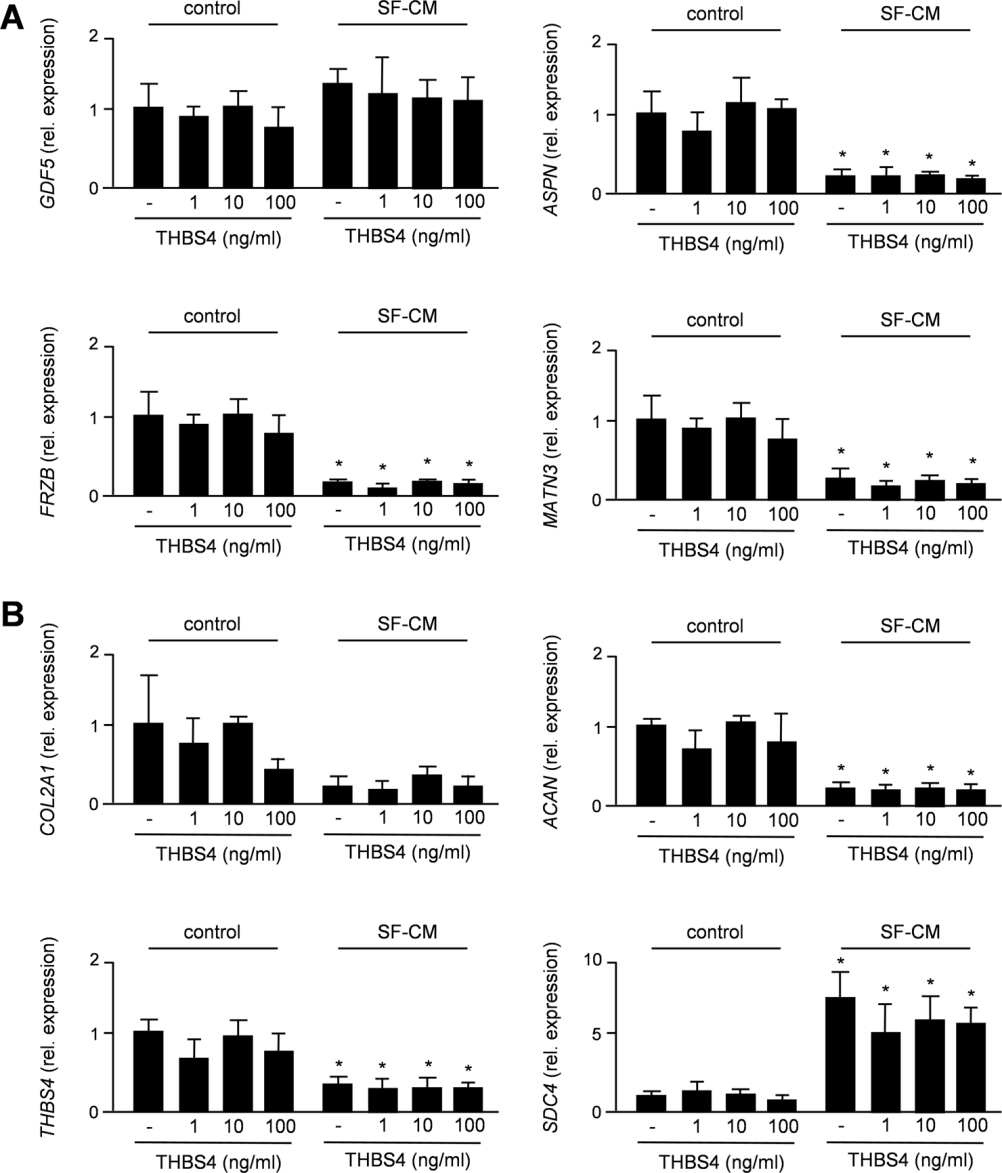
THBS4 does not affect gene expression in porcine articular chondrocytes. (A) qRT-PCR monitoring OAS gene expression in porcine articular chondrocytes treated with human THBS4 and/or SF-CM for 6 hours. (B) qRT-PCR monitoring expression of *COL2A1*, *ACAN*, *THBS4 and SDC4* expression in the same samples. Bars represent mean ± SD (n = 4). Asterisks indicate statistically significant differences towards untreated cells (p<0.05).

As we observed reduced expression of *Aspn* in articular cartilage of 26 weeks old *Thbs4*-deficient, we additionally treated articular chondrocytes for 6 hours with THBS4 and/or SF-CM, before isolating RNA for qRT-PCR expression analysis. When monitoring expression of the OAS genes we observed no significant influences of THBS4, either alone or in the presence of SF-CM (Fig 8A). Importantly however, expression levels of *ASPN*, *FRZB* and *MATN3 (*but not of *GDF5)* were remarkably reduced by SF-CM, thus suggesting a direct transcriptional influence by yet unidentified SF-derived molecules. Based on these findings we additionally monitored expression of *COL2A1*, *ACAN*, *THBS4* and *SDC4*, the latter gene encoding a negative regulator of articular chondrocytes [9]. Again, we failed to detect a significant influence of THBS4, either alone or in the presence of SF-CM (Fig 8B). In contrast, whereas SF-CM caused a transcriptional repression of *ACAN* and *THBS4*, *SDC*4 expression was more than 5-fold induced by SF-CM, thus underscoring the suspected influence of SF-derived molecules on gene expression in articular chondrocytes. With respect to THBS4 however, our combined results suggest that it does not act as a signaling molecule directly regulating articular proliferation, differentiation or gene expression.

## Discussion

The thrombospondins represent a family of secreted matricellular proteins potentially regulating various processes of tissue remodeling [31]. The five Thbs family members can be divided into two subgroups based on their domain structure and mode of multimerization. Thbs4, together with Thbs3 and Thbs5, belongs to the second subgroup considered to form a pentameric stucture [32]. While mutations of Thbs5, better known as cartilage-oligomeric matrix protein (Comp), cause two different forms of skeletal dysplasia, the role of Thbs3 and Thbs4 in the skeleton are still poorly defined [33,34]. More specifically, while a transiently accelerated endochondral ossification has been reported for mice lacking Thbs3, the skeletal phenotype of *Thbs4*-deficient mice has not been analyzed previously. Interestingly however, two recent studies have identified a specific function of Thbs4 in myocardial remodeling [35,36], thereby underscoring the relevance of previous findings showing that *Thbs4* expression is specifically induced in hypertrophic or failing hearts [37,38]. More recently, *Thbs4*-deficient mice were found to display an altered composition of extracellular matrices in tendons and skeletal muscles, which also affected the physiological functions of both tissues [39].

Since we have previously identified Thbs4 as a marker of articular cartilage [15], our main interest was an in-depth skeletal phenotyping of *Thbs4*-deficient mice, thereby also addressing the question, whether its increased expression in bones from *Hyp* mice is relevant for the pathogenesis of X-linked hypophosphatemic rickets [24]. Through the use of undecalcified histology with subsequent histomorphometry we found that *Thbs4*-deficiency has no impact on skeletal growth, bone mass acquisition or skeletal remodeling, and we were able to rule out a contribution of Thbs4 to the skeletal phenotype of Phex-deficient *Hyp* mice. We did however observe a significant reduction of articular cartilage thickness in 26 weeks old *Thbs4*-deficient mice when compared to wildtype littermates, although there was no difference found in 6 or 52 weeks old animals. More specifically, it appeared that the age-related gain of articular cartilage thickness is abolished in *Thbs4*-deficient mice, whereas the ageing-associated articular cartilage degeneration was not accelerated [40–42]. These data indicate that Thbs4 has a protective role for articular cartilage integrity and suggest that its absence is partially compensated by other molecules, possibly Thbs family members, thereby preventing complete loss of joint surfaces in *Thbs4*-deficient mice. We additionally crossed the *Thbs4*-deficiency into a TNF-transgenic background, which is commonly used to study the impact of specific molecules on the severity of rheumatoid arthritis [16,43,44]. Here we did not observe a significant impact of the *Thbs4*-deficiency on two clinical scores of rheumatoid arthritis, i.e. paw swelling and grip strength, yet loss of subchondral bone was apparently enhanced in *Thbs4*-deficient TNF-transgenic mice, thereby supporting the concept that Thbs4 has a protective role in articular cartilage.

With respect to the underlying molecular mechanisms we performed experiments with porcine articular chondrocytes, thereby avoiding the principal problem to obtain primary murine articular chondrocytes at sufficient quantity and without contaminating additional cell types. Here we administered recombinant human THBS4 to study its potential effects on different cellular parameters. Using the porcine system additionally allowed us to introduce a control, i.e. conditioned medium from synovial fibroblasts (SF-CM), since these cells appear to secrete factors modulating activities of articular chondrocytes [45]. We found, unexpectedly, that short-term treatment with SF-CM significantly increased Caspase-3/7 activity and *SDC4* expression in articular chondrocyte cultures, while it reduced the expression of genes associated with osteoarthritis and/or encoding components of the cartilage extracellular matrix, including *THBS4*. Albeit interesting and worth being further investigated, the most important finding related to the present study however was that THBS4 administration did not affect any of the tested parameters, and it did not protect against the negative influence of SF-CM. Therefore, although we observed a specific reduction of *Aspn* expression in 26 weeks old *Thbs4*-deficient mice, it is unlikely that this alteration is directly caused by *Thbs4*-deficiency, since our combined analyses essentially rule out that THBS4 acts as a signaling molecule directly regulating transcription in articular chondroctes. We additionally performed cell adhesion assays, thereby confirming that COMP/THBS5 mediates chondrocyte attachment in an integrin-dependent manner [30], unlike THBS4. Albeit these findings are principally consistent with the lack of differences regarding cellular density in articular cartilage between wildtype and *Thbs4*-deficient mice, they failed to provide a molecular explanation for the observed differences. Therefore, we can only speculate about the causes of the transient reduction of articular cartilage thickness in *Thbs4*-deficient mice. However, since Thbs4 has been shown to interact with various matrix molecules [46], this phenotype might be related to subtle differences in extracellular matrix integrity, similar to the tendons, where *Thbs4*-deficiency affects collagen fibrillogenesis [39].

The absence of evidence supporting a function of THBS4 as a signaling molecule essentially rules out the possibility that the development of drugs activating THBS4 is a possible approach for the treatment of osteoarthritis. However, since *THBS4*, in contrast to the other THBS family members or additional matrix proteins, such as type-II-collagen or aggrecan, is specifically expressed by articular and not by growth plate chondrocytes, monitoring *THBS4* expression could still be useful for the quality control of tissue-engineered articular cartilage [15]. In the same line of thought, it is reasonable to hypothesize that THBS4 or THBS4 fragments could serve as biomarkers to monitor joint destruction. In fact, while it is obvious that the introduction of disease-specific biomarkers, such as PSA for prostatic hyperplasia, has revolutionized disease management in the last decades, a screening or treatment monitoring for osteoarthritis is still not possible, since specific markers of articular cartilage remain to be identified [47]. We therefore measured THBS4 serum concentrations in individuals with mono-osteoarthritis (S2A Fig) or poly-osteoarthritis (S2B Fig) using a commercially available ELISA against intact THBS4. Although we found that the latter group displayed higher circulating levels of intact THBS4, the difference towards individuals with mono-osteoarthritis or controls was not significant (S2C Fig). This implies that THBS4 is most likely not a valid biomarker of human osteoarthritis, yet it might be useful to analyze a larger number of individuals, also including cases with other causes of articular cartilage loss (such as rheumatoid arthritis), and to analyze for the presence of THBS4 cleavage products that are potentially generated in specific pathological settings.

## Supporting Information

S1 Fig Loss of subchondral bone in TNF-transgenic *Thbs4*-deficient mice. (A) Quantification of foot paw swelling (left) and grip strength (right) over time in TNF-transgenic mice with (WT) or without (KO) a functional *Thbs4* allele. Values represent mean ± SD (n = 4 per group). (B) Von Kossa/van Gieson staining of knee joints from 12 weeks old TNF-transgenic mice with (+/+) or without (-/-) a functional *Thbs4* allele. The quantification of the subchondral bone volume is given on the right. Bars represent mean ± SD (n = 4 per group). Asterisks indicate statistically significant differences between WT and KO (p<0.05).
(TIF)

S2 Fig THBS4 concentrations in sera from individuals with osteoarthritis. (A) Age and gender distribution of individuals with mono-osteoarthritis (n = 20). (B) Age and gender distribution of individuals with poly-osteoarthritis (n = 21). (C) THBS4 concentrations in the sera from patients with mono-osteoarthritis (mOA) or poly-osteoarthritis (pOA). The dotted red line indicates the mean serum concentration measured in 6 control individuals without osteoarthritis.
(TIF)
