# Supplementary figures and images for: Deficiency of Thrombospondin-4 in Mice Does Not Affect Skeletal Growth or Bone Mass Acquisition, but Causes a Transient Reduction of Articular Cartilage Thickness
Source: PLoS One. 2015 Dec 2;10(12):e0144272. doi: 10.1371/journal.pone.0144272 (PMC4667928; doi:10.1371/journal.pone.0144272)

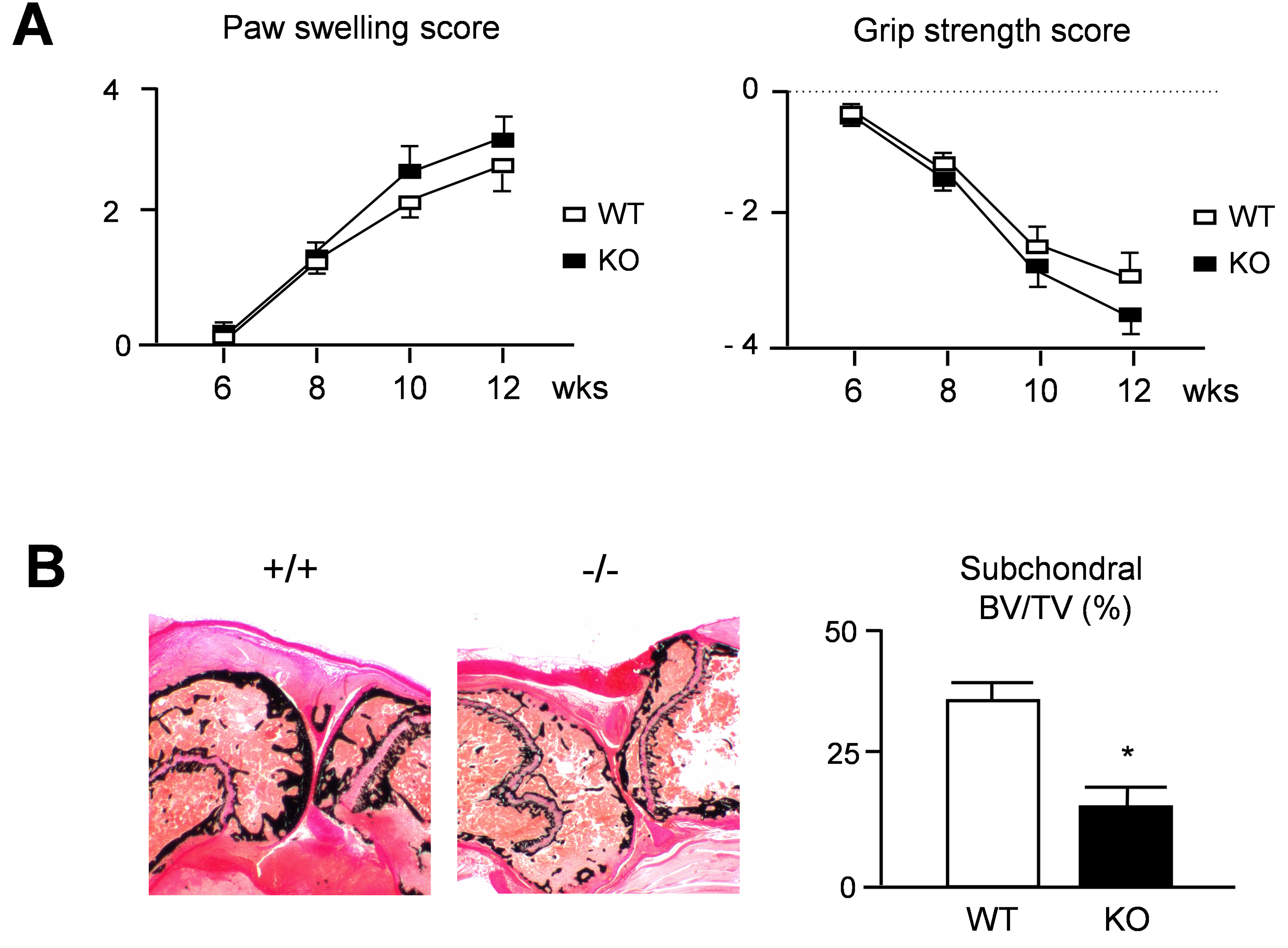

Supplement: S1 Fig — (A) Quantification of foot paw swelling (left) and grip strength (right) over time in TNF-transgenic mice with (WT) or without (KO) a functional Thbs4 allele. Values represent mean ± SD (n = 4 per group). (B) Von Kossa/van Gieson staining of knee joints from 12 weeks old TNF-transgenic mice with (+/+) or without (-/-) a functional Thbs4 allele. The quantification of the subchondral bone volume is given on the right. Bars represent mean ± SD (n = 4 per group). Asterisks indicate statistically significant differences between WT and KO (p<0.05). (TIF) [file pone.0144272.s001.tif]

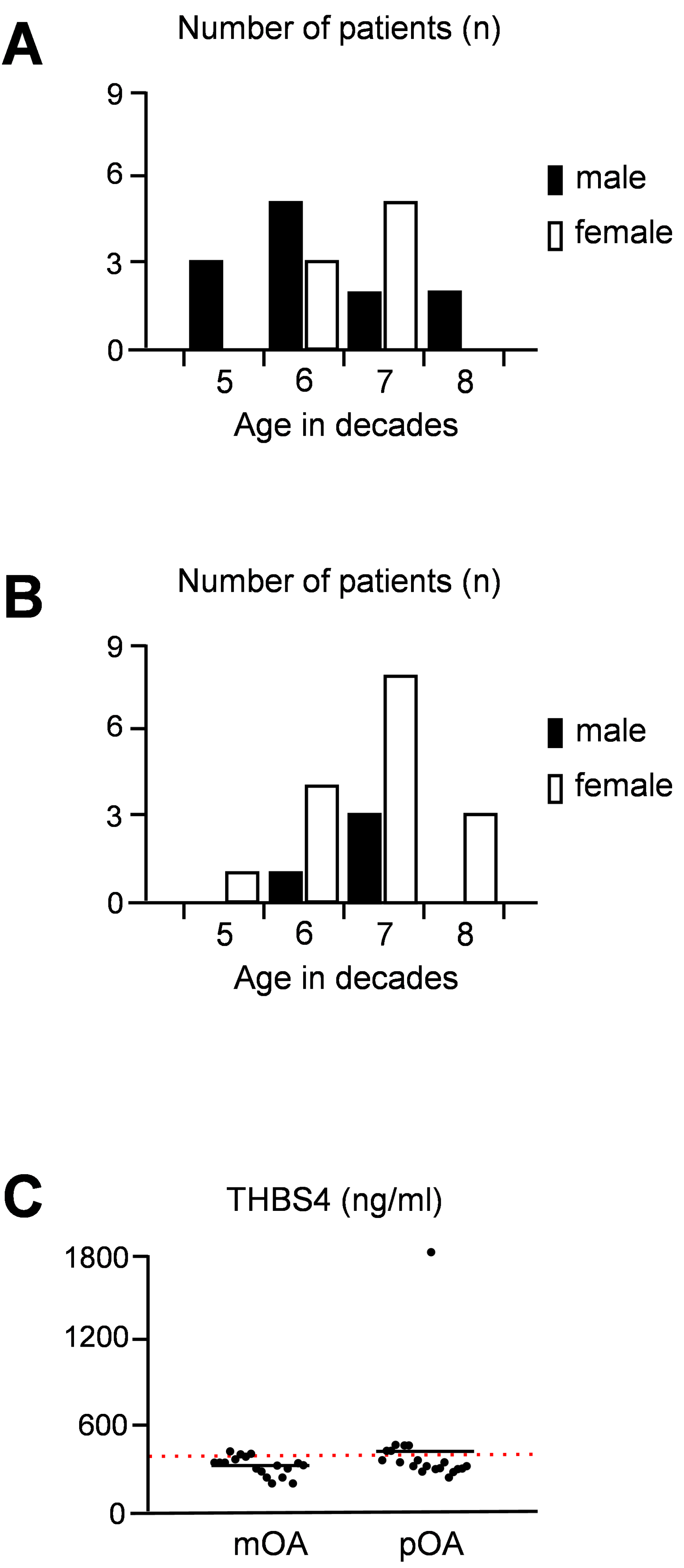

Supplement: S2 Fig — (A) Age and gender distribution of individuals with mono-osteoarthritis (n = 20). (B) Age and gender distribution of individuals with poly-osteoarthritis (n = 21). (C) THBS4 concentrations in the sera from patients with mono-osteoarthritis (mOA) or poly-osteoarthritis (pOA). The dotted red line indicates the mean serum concentration measured in 6 control individuals without osteoarthritis. (TIF) [file pone.0144272.s002.tif]
